# Editorial: Preventing obesity-related degenerative diseases through lifestyle changes

**DOI:** 10.3389/fspor.2026.1808530

**Published:** 2026-03-04

**Authors:** Shengdi Lu, Yun Shen, Gang Hu, Ding Jian, Chen Wang

**Affiliations:** 1 Chronic Disease Epidemiology Laboratory, Pennington Biomedical Research Center, Baton Rouge, LA, United States; 2 Department of Orthopaedics, Shanghai Sixth People’s Hospital, Shanghai Jiao Tong University, Shanghai, China; 3 Department of Orthopaedics, National University Hospital, Singapore, Singapore

**Keywords:** degenerative diseases, digital health, lifestyle interventions, obesity, physical activity, prevention, sedentary behavior, sleep

## Introduction

Obesity is increasingly recognized as a chronic, relapsing disease arising from complex interactions among biological susceptibility, behaviors, and the wider environment (1). Beyond its effects on body weight, obesity contributes to the burden of noncommunicable diseases and long-term disability (1). WHO reports that in 2022, 1 in 8 people worldwide were living with obesity; 890 million adults were living with obesity; and adult obesity has more than doubled since 1990 (1). As countries face continued increases in obesity prevalence and downstream burden in health systems and communities, prevention-oriented strategies that are feasible, scalable, and acceptable to diverse populations become increasingly urgent (1).

The Research Topic “*Preventing obesity-related degenerative diseases through lifestyle changes*,” published in Frontiers in Sports and Active Living (section: *Physical activity in the prevention and management of disease*), was conceived in direct response to this challenge (2). The Topic scope highlights that obesity-related degenerative diseases, including type 2 diabetes, cardiovascular diseases, and osteoarthritis, are influenced by multiple, interacting lifestyle domains (2). It emphasized lifestyle interventions that can mitigate immediate health consequences while also addressing longer-term complications, and it welcomed contributions spanning exercise interventions, structured vs. unstructured activity patterns, digital health tools, telehealth and remote coaching, community programs and policy interventions, and complementary exercise approaches such as tai chi (2). The final collection comprises five Original Research articles published across the Topic, including work in Frontiers in Sports and Active Living (3).

Specifically in this Research Topic, Chiang et al. investigated an epigenetic correlate of lifestyle behavior by analyzing Taiwan Biobank data and reporting an association between tai chi participation and higher DNA methylation levels at the IL20 promoter compared with non-exercisers Chiang et al. While the cross-sectional design precludes causal inference, the study is noteworthy for connecting a low-impact, accessible movement modality to a biologically plausible signal related to immune and inflammatory pathways. In the context of obesity-related degeneration, where chronic low-grade inflammation is frequently discussed as a contributory mechanism, this work illustrates one way in which lifestyle behaviors may be interrogated not only through questionnaire-reported exposures and clinical outcomes, but also through molecular and epigenetic signatures relevant to mechanism and hypothesis generation Chiang et al.

Lee et al. addressed lifestyle change under real-world constraint by examining associations between COVID-19–related daily life restrictions, self-reported behavior changes, and obesity level in Korean adults using the 2020–2021 Korean Community Health Surveys Lee et al. Their results indicate that restrictions and unhealthy behavior changes, including decreased physical activity and increased fast-food consumption, progressively increased with obesity severity, with sex-stratified differences in associations Lee et al. Beyond the immediate pandemic context, these findings underscore the importance of prevention systems that can maintain and support healthy behaviors during social disruption. They also highlight that “lifestyle change” is not merely an individual-level decision, but is patterned by context, constraints, and access, considerations that are central when designing scalable interventions and policies intended to reduce degenerative disease risk in populations living with obesity Lee et al.

Two additional studies contribute tools and quantitative frameworks that can support earlier and more actionable prevention. Jiang and He developed an interpretable, lifestyle-based obesity prediction model for Chinese adolescent students using an anonymized dataset incorporating family factors (including parental BMI), diet-related behaviors, sleep duration, and physical fitness indicators Jiang and He. By prioritizing interpretability alongside predictive performance, the model is positioned as a screening-support tool that can better inform targeted health education and resource allocation, particularly in school settings where low-cost, scalable approaches are necessary for early prevention Jiang and He. Complementing this risk-identification approach, Torres-Carballo et al. applied isotemporal substitution modeling to explore how reallocating time among sedentary behavior, physical activity intensities, and sleep relates to body composition among individuals with prediabetes and overweight/obesity Torres-Carballo et al. Their findings suggest that replacing sedentary time with moderate-to-vigorous physical activity, rather than with light activity or sleep, was associated with more favorable adiposity-related measures, providing a useful quantitative framework for time-use tradeoffs when counseling patients and designing interventions Torres-Carballo et al.

Finally, Curovic and Grecic foregrounded delivery systems and workforce capability as determinants of whether lifestyle interventions are implemented effectively and sustained Curovic and Grecic. Through qualitative interviews with high-level personal trainers in Serbia, the authors described professional development pathways alongside perceived gaps in education and regulation, including the absence of national accreditation structures and limited formal training in behavior change models and inquiry-based skills Curovic and Grecic. Their work argues for a shift from performance-centric approaches toward more holistic, person-centered practice, explicitly linking professional preparation to the quality, safety, and sustainability of lifestyle interventions. In doing so, the study highlights an increasingly important bridge between evidence and everyday practice: the competencies, training pathways, and support structures of exercise professionals who often deliver behavior change “on the ground” Curovic and Grecic.

Taken together, these five studies advance the broader field by illustrating why preventing obesity-related degenerative diseases through lifestyle change must operate across levels, from plausible mechanisms, to population behavior under constraint, to interpretable prediction and early targeting, to quantitative time-use tradeoffs, and to scalable delivery capacity in practice (3, Chiang et al., Lee et al., Jiang and He, Torres-Carballo et al., Curovic and Grecic). The collection aligns with WHO guidance that emphasizes both increasing physical activity and reducing sedentary behavior across populations and abilities, including among people living with chronic conditions (Chiang et al., Lee et al.). In practical terms, the studies collectively move the field from generic “move more” messaging to more implementation-relevant questions: which modalities and intensities matter, how time is redistributed in real days, how risk can be identified early, and how practitioner systems can support sustained behavior change.

Looking forward, priorities for research and practice include longitudinal and intervention designs that test causal pathways and durability of effects, and multi-component lifestyle programs that address activity, sedentary time, diet quality, sleep regularity, and stress-related behaviors in combination rather than in isolation (2). Implementation research is equally critical, including studies of digital health and telehealth delivery models highlighted in the Topic scope, strategies to reduce structural barriers to healthy eating and movement, and professional training models that equip exercise practitioners with evidence-based behavior change competencies. Taken together, these directions underscore a central message of the Research Topic: preventing obesity-related degenerative diseases through lifestyle change is scientifically plausible and clinically meaningful, but success will depend on interventions and systems that are both effective and sustainable across the life course.
